# “PrEP a double-edged sword”: Integrating implementation science methodology with Photovoice to guide culturally-tailored pre-exposure prophylaxis (PrEP) programs for Latino/a and non-Latino/a men who have sex with men in South Florida

**DOI:** 10.1371/journal.pone.0305269

**Published:** 2024-08-09

**Authors:** Ariana L. Johnson, Kyle J. Self, Rebe Silvey, Gabrielle A. Webb, Nonie Kalra, Stephen Fallon, Suzanne M. Randolph Cunningham, Mariano Kanamori

**Affiliations:** 1 Department of Public Health Sciences, University of Miami, Coral Gables, Florida, United States of America; 2 University of Miami, School of Education and Human Development, Coral Gables, Florida, United States of America; 3 Latinos Salud, Miami, Florida, United States of America; 4 The MayaTech Corporation, Silver Spring, Maryland, United States of America; David Geffen School of Medicine at UCLA, UNITED STATES

## Abstract

**Background:**

DiversiPrEP is a culturally-tailored PrEP program for LMSM offered in South Florida. DiversiPrEP navigates LMSM through their PrEP journey, including education, deciding if PrEP is relevant for them, payment, and accessing/maintaining PrEP use. DiversiPrEP includes five ERIC strategies (Increase Demand, Promote Adaptability, Alter Client Fees, Intervene with Clients to Enhance Uptake and Adherence, and Tailor Strategies).

**Description:**

Photovoice was used to conduct five two-part focus groups with LMSM (n = 12) and Non-LMSM (n = 12). In the first session, trainers provided guidance on selecting and contextualizing photos to generate CFIR themes. Then, participants captured photos that embodied their lived experiences accessing PrEP. In the second session, using SHOWeD, participants discussed photos, identifiedhow photos relate to culturally relevant issues. Triangulation approaches compared/contrasted themes between LMSM and Non-LMSM.

**Results:**

Five central themes emerged around barriers and facilitators to PrEP services: 1) the need for normalizing PrEP messages within the MSM community, 2) the need for normalizing PrEP messages outside the MSM community, 3) the need for expanding PrEP knowledge, 4) different motivations for using PrEP, and 5) the presence of structural barriers that limit PrEP access. This study compared similarities and differences of barriers and facilitators to PrEP use between Latino/a and non-Latino/a MSM. Similarities included the built environment (*outer setting)* as a barrier, the need for normalizing PrEP messaging within and outside of the MSM community, and the need to expand PrEP knowledge. Differences between Latino/a and non-Latino/a MSM were found in assessing the motivation and personal drivers (*inner setting*) for initiating PrEP associated with how participants viewed their responsibilities to self or others.

**Conclusions:**

Photovoice with focus groups identified CFIR constructs that can guide the large-scale implementation of a client-centered PrEP service model with telehealth for both Latino/a and non-Latino/a MSM. Implementing client-centered accessible PrEP programs is an essential step to promoting sexual-health equity.

## Background

It is estimated that men who have sex with men (MSM) account for approximately 3.9% of the total population in the United States[1]. Despite being a U.S. minority population, MSM make up the majority of new HIV diagnoses, at an alarming 69% [2]. Prevention treatment, known as Pre-Exposure Prophylaxis (PrEP), have been approved by the FDA in both oral and injectable forms in 2016 and 2021, respectively and reduce HIV transmission by about 99% when taken as prescribed [3,4]. However, there is still a tremendous gap in access for those at risk for HIV; in 2020, only 25% of the 1.2 million individuals eligible for PrEP were given a prescription [3,5]. Coverage is not equal across racial and ethnic groups, with Black and Latino/a people making up nearly 70% of new HIV infections in the United States (i.e., more than 40% and about 29%, respectively); yet, White people made up 60% of the prescriptions for PrEP in 2019 [6].

Patients’ ability to initiate and maintain taking PrEP that requires overcoming personal and systemic barriers including structural barriers (e.g., discrimination, poverty/financial stress) concerns over side effects of PrEP, effects of PrEP, and personal recommendations for treatment; barriers that are especially difficult to overcome for underserved populations such as Latino/as [7,8]. One way of identifying these barriers for populations that are disproportionally impacted by systemic healthcare inequities is through utilizing an implementation science framework. Implementation science allows systematic tailoring of interventions by identifying determinants of practice, designing implementation interventions tailored to determinants, and implementing and evaluation implementation interventions based on identified determinants [9,10]. Evaluating the effectiveness in a specific setting identifying barriers at multiple levels can be systematically completed by following a Consolidated Framework for Implementation Research (CFIR) [11]. When using a framework like CFIR, determinants are located in the first left column of the logic model and are mapped to CFIR constructs that are relevant to the implementation project. The CFIR provides operationally defined constructs from multiple disciplinary domains that are likely to influence the implementation of PrEP service programs, which are broken down into five major domains that includes the outer setting (ex. Patient needs and resources), inner setting (ex. Compatability), characteristics of individuals involved (ex. Knowledge and attitudes), process (ex. Quality and extent of planning), and characteristics of the program (ex. Evidence strength and quality). The CFIR domain and constructs can help understand and evaluate social, structural, and logistical determinants acting as barriers or facilitators in the implementation and sustainability of a PrEP Program. However, many CFIR approaches often rely on traditional data collection methods such as interviews and focus groups and often only use CFIR constructs to guide data analysis, which may limit engagement of community participants who are the potential users of the interventions [12].

To address this limitation when focusing research on underserved populations is to identify these determinants through integration of implementation science methodology with methods that increase community engagement in data collection. For this study, we integrated CFIR-based focus group methodology with Photovoice. Photovoice is a Community-Based Participatory Research (CBPR) process that empowers participants to identify, represent, and discuss issues from their perspective using photography. Community members are gathered to identify facilitators and barriers of an important community issue by taking photos and discussing the findings to present to stakeholders [13]. Participants take photos independently, then meet as a group to engage in dialogue related to the photos that represent phenomena that can influence their health decisions and wellbeing. Photovoice is particularly useful for implementation science projects addressing sensitive and stigmatized topics because it enables a safe platform to share their lived experiences through dialogue and visual media [14–16]. As such, Photovoice is a well-suited method for talking about HIV prevention, including PrEP [14,16–19].

CFIR has been shown to be useful in identifying key determinants (i.e., barriers and facilitators) to adoption and scaling up interventions [20,21]. This study integrated the CFIR framework with Photovoice to stimulate participant discussions about barriers and facilitators of effective implementation of PrEP programs for Latino/a and non-Latino/a MSM groups that fall within various CFIR domains [14–18,22–24]. The goal of this study was to identify modifiable factors within three of the CFIR domains: the *outer setting* (e.g., Latino/a cultural values), *characteristics of the intervention* (e.g., adaptations for the local setting), and *characteristics of individuals* (e.g., awareness, beliefs, intentions, accessibility) for successful implementation of PrEP programs for Latino/a and non-Latino/a MSM.

## Methods

Semi-structured qualitative discussions were conducted using focus groups paired with Photovoice. These semi-structured interview guides were created using cfirguides.org to map questions with CFIR constructs. Alongside the semi-structured interview guide, we incorporated Photovoice’s SHOWeD method to stimulate group discussion through a set of six guiding questions [15,16].SHOWeD complements and enhances Photovoice by providing a semi-structured guideline to describe each photograph presented, help participants in identifying the social, structural, and political barriers and facilitators, and finally develop potential intervention strategies [15]. Participants described their photo, identified the importance and relevance of what was seen in the photo, and the actions that could be taken to eliminate barriers and promote access to treatment [19]. This study is reported according to procedures adapted from the consolidated criteria for reporting qualitative (COREQ) research [25].

### Participants

Recruitment was facilitated through our community partner, a community-based organization (CBO) that focuses services on health education and integrated HIV prevention and screening for MSM in South Florida. Recruitment and enrollment took place between June 3^rd^, 2021 and August 8^th^, 2022. All clients who received any in-person services at the CBO during the 13-month recruitment period were informed about the study and invited to screen for eligibility during their appointment. Those who expressed interest were then screened for eligibility (described below); those who did not meet minimum eligibility were informed they could no longer move forward with enrollment. The CBO and University of Miami have a successful track record of collaboration spanning the past 9 years and work collaboratively in a mutual effort to end the HIV epidemic. To be a participant in this study, individuals had to be 18 years or older, meet the CDC clinical guidelines for PrEP eligibility, speak English or Spanish, have access to a cellphone that could take pictures, and have access to a computer with internet capabilities to participate in online meetings. We originally enrolled 30 individuals, with 6 dropping out between focus group session 1 and focus group session 2. Demographics for the enrolled 30 can be found in Table 1. There were twenty-four participants who completed both sessions (12 Latino/a MSM and 12 non-Latino/a MSM). This study was implemented in Miami-Dade and Broward Counties, where the CBO has three locations from which recruitment was conducted. These counties have the highest population of any county in Florida and continue to hold the highest number of individuals diagnosed with HIV in the state [6,26]. As of 2018, Florida ranked third in the United States for highest rates for new HIV diagnoses; thus, the Florida Department of Health identified the reduction of transmission of HIV and HIV-related deaths as one of the seven public health priority goals [6,26]. The proportion of new HIV cases amongst Latino/a has risen most rapidly in these counties over the last 10 years, from 50% to 68% in Miami-Dade, and from 18% to 31% in Broward. As a result, these counties’ top priority populations for HIV prevention were Latino/a MSM [6,26].

**Table 1:** 

	Overall (N = 30)
**Age**	
Mean (SD)	29.6 (5.92)
Median [Min, Max]	27.0 [22.0, 42.0]
**Ethnicity**	
Non-Hispanic	12 (40.0%)
Hispanic/ Latino	18 (60.0%)
**Race**	
African American	6 (20.0%)
Afro-Caribbean	2 (6.7%)
American Indian/Alaskan	1 (3.3%)
Asian/Pacific Islander	1 (3.3%)
Multi-racial	4 (13.3%)
Prefer not to answer	2 (6.7%)
White, Hispanic	6 (20.0%)
White, Non-Hispanic	8 (26.7%)
**Employment**	
Full time—over 30 hours per week	6 (20.0%)
Part time—less than 30 hours per week	6 (20.0%)
Stay at home	7 (23.3%)
Student	5 (16.7%)
Never worked	2 (6.7%)
Missing	4 (13.3%)
**Income**	
Less than $4,999	6 (20.0%)
$25,000 - $34,999	6 (20.0%)
$35,000 - $49,999	7 (23.3%)
$50,000 - $74,999	5 (16.7%)
$75,000 - $99,999	2 (6.7%)
Missing	4 (13.3%)
**Sex with male**	
2–6 months	3 (10.0%)
Past month	27 (90.0%)
**HIV Status**	
HIV-negative	30 (100%)

#### Procedures

Each of the three facilities of our community partner had a designated coordinator that served as a liaison to our study team for facilitating recruitment and implementation. These coordinators participated in the study design process and met weekly with the University of Miami study team to discuss project goals, share feedback, and ensure continued communication. We also implemented a virtual two-hour train-the-trainer session for coordinators, which was aimed at modeling their role as facilitators of the participant Photovoice and focus group sessions. After completion of the train-the-trainer session, we implemented five Photovoice discussions which consisted of two sessions, which were implemented virtually to comply with COVID-19 pandemic restrictions. All sessions were audio-recorded with participants’ permission to accurately capture discussion content. Each session included up to six participants, one facilitator from the University of Miami, and the assigned coordinator. The facilitator is lead author of this manuscript (female sex assigned at birth), who was a doctoral student at the time of the study, and had been extensively trained in both implementation science research and Photovoice by experts in the field prior to involvement in this project. There were two non-Latino/a Photovoice groups with six participants (n = 12) and three Latino/a Photovoice groups with four participants (n = 12).

Prior to the start of the first online session, the informed consent was read allowed while each participant was asked to follow along. Any and all questions were addressed. Consent was then electronically signed via REDCap by each participant prior to starting the first session and a PDF was provided for each. During the first session, participants were introduced to the primary goals of the project, the facilitator, and the desire to publish a manuscript with the findings. The facilitators discussed her interest and passion for HIV prevention. They were then taught the basics of good photography, ethical photography principles, and instructed on the Photovoice assignment. The Photovoice assignment would be guided by two prompts to stimulate discussion: (1) What are the greatest barriers to PrEP services? (2) What are the greatest facilitators to PrEP services? These prompts were developed in collaboration with the Implementation Science Coordination Initiative (ISCI), responding to the constructs assessing context and tailoring strategies. At the first session, participants were asked to consider these prompts and take photographs independently on their phone in their daily lives that represented their views and experiences related to the prompts for next two weeks. After taking five photographs during the two weeks between session one and two, each participant uploaded them securely to REDCap. Prior to beginning session two, each participant was asked individually in a breakout room if they were comfortable sharing their captions and opinions; if they were comfortable with doing so, the facilitator presented a slideshow containing all participants’ photos, with the aim to generate a caption for each photo that would highlight CFIR-related determinants (i.e., barriers and facilitators). At that time in the focus group with other participants, each participant was asked to say a few sentences about each of the photographs.

In-depth discussions of each photo were facilitated using the SHOWeD method that involved: (1) discussing a picture literally; (2) identifying how a picture relates to relevant issues; and (3) developing actions that could be taken to address those issues[27]. The SHOWeD questions were based on the letters in the acronym and included: “What do you *S**ee* here? What is really *H**appening*? How does this relate to *O**ur* lives? *W**hy* does this problem or strength exist? How can we become *E**mpowered* through this new understanding? What can we *D**o* about it?” Participants incorporated answers to each of these questions in their caption when describing their photos. Upon completion of the captioning, participants voted on the top five photos that they felt best captured the barriers and facilitators to PrEP services they had identified. Then, once a group reached consensus, the team developed a list of barriers and facilitators to PrEP services and used this list to guide discussions on how each theme applied to both Latino/a and non-Latino/a MSM communities. These lists were then provided to the research team for analyses.

#### Ethics

This study was approved by the University of Miami Institutional Review Board. Participants received a $35 gift card after participating in session one and a $40 gift card after participating in session two. Prior to the start of the first online session, the informed consent was read allowed while each participant was asked to follow along. Any and all questions were addressed. Consent was then electronically signed via REDCap by each participant prior to starting the first session and a PDF was provided for each [28,29]. This was not a clinical trial.

### Data analysis

Triangulation approaches were used to compare the list of themes and issues identified by participants across sites and subpopulations [30]. Our analysis took place over four phases. In phase 1, descriptive observational field notes, SHOWeD captions, and themes provided by participants were used by the research team to create preliminary sub-categories. In phase 2, preliminary sub-categories and transcribed text from the interviews were analyzed using qualitative content analysis to describe what was perceived as a barrier and what was perceived as a facilitator[31]. The description and text provided by the participants remained as close to the spoken and written text provided by them. In phase 3, the research team (consisting of four graduate students and one implementation science expert) reviewed the relationships among subcategories across focus groups, and then combined related subcategories into broader categories which resulted in the creation of central themes. Findings from the existing literature and CFIR domains were used to review and refine central themes [16,20,32,33]. The final set of central themes, the CFIR domains that these themes related to, and the corresponding captioned photographs constituted preliminary findings. In phase 4, these sets of themes and captioned photographs were sent back to each participant to ensure that the description and context appropriately represented what they had discussed. Suggested changes from participants were then incorporated into the final list of coded themes (barriers and facilitators) and captioned photographs.

## Results

The following five central themes emerged around barriers and facilitators to PrEP services for Latino/a and non-Latino/a MSM in South Florida: 1) the need for normalizing PrEP messages within the MSM community, 2) the need for normalizing PrEP messages outside the MSM community, 3) the need for expanding PrEP knowledge, 4) different motivations for using PrEP, and 5) the presence of structural barriers that limit PrEP access. These themes, their associated images, and captions were chosen directly by the focus group participants. Of the 31 CFIR constructs assessed the central themes and associated quotes guided by focus group input were best mapped to 4 CFIR constructs: 1) Knowledge & Beliefs about the Intervention, 2) Access to Knowledge and Information, 3) Needs & Resources of Those Served by the Organization, and 4) Available Resources. A table of the mapped themes and CFIR constructs can be found in S1 Table.

### Knowledge & beliefs about the intervention

Perceptions of attitudes surrounding PrEP messaging was expressed under the CFIR construct ‘knowledge and beliefs about the intervention’, from the perspective of individuals involved in the focus groups. This construct distinguished between MSM and non-MSM communites.

### Normalizing PrEP messages within the MSM community

Within the MSM community participants highlighted the importance of having access to PrEP information and information to overcome the stigma surrounding HIV testing. One Latino/a focus group participant shared that:

“Individuals in the community may not know their status because they do not ‘hook up’ or engage in sex with random people and because of this they do not get tested or take PrEP.”

**Fig 1:**
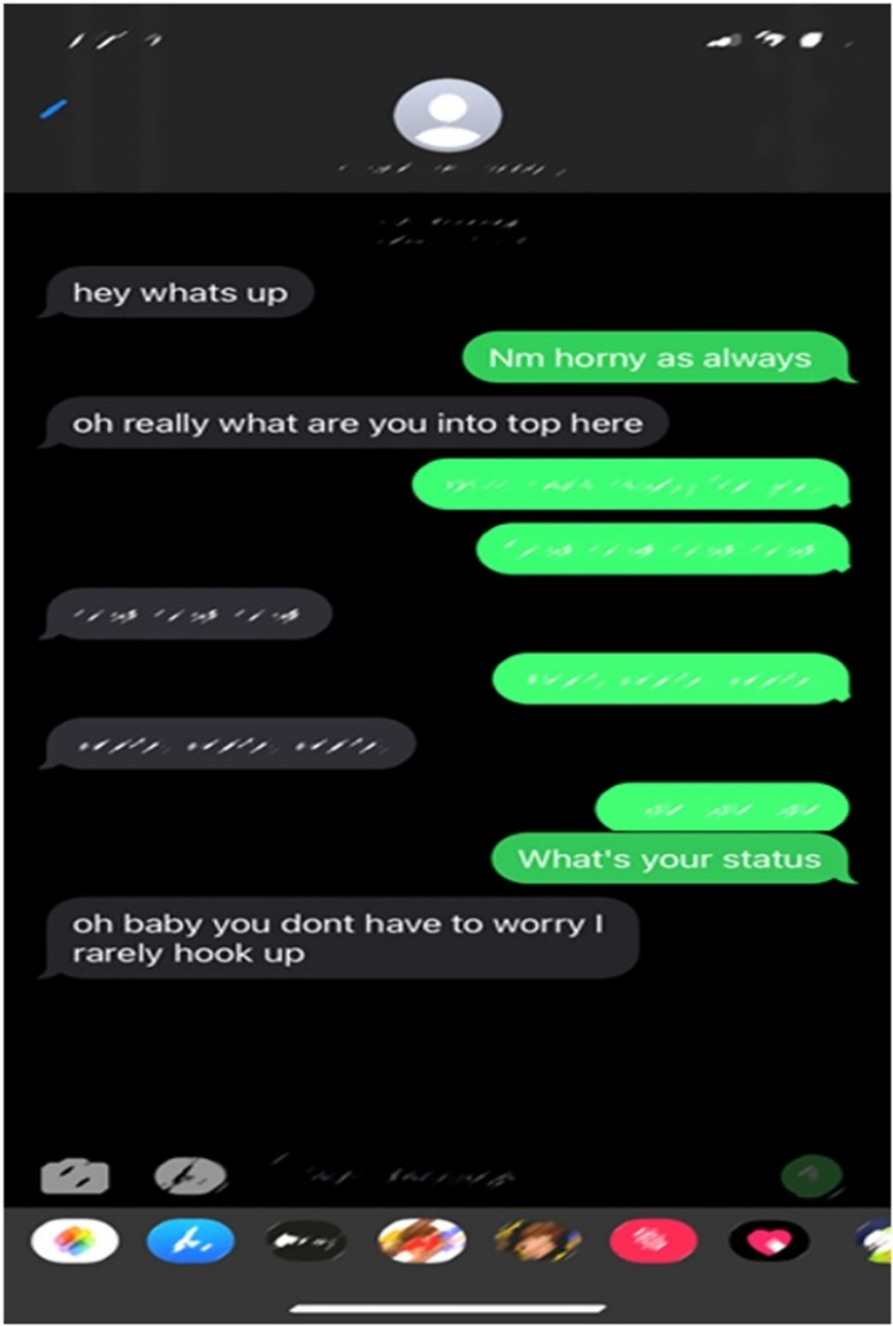
A text exchange between an individual and their potential sexual partner.

A participant’s photo of a text exchange between potential sexual partners (Fig 1) described the current lack of knowledge about PrEP services available to the Latino/a MSM community. Participants also mentioned that this photo was a good representation of PrEP stigma—the perception that PrEP users are more promiscuous than non-PrEP users. A group of Latino/a participants identified this stigma as a barrier to PrEP access. They also identified that increasing access to PrEP information was necessary to overcome this barrier:

“Access [to information] is everything. When opening your apps like Grindr, Scruff and others they advertise for different PrEP services in your community or online like Qcare+ or HeyMistr it makes the process seem more accessible and visible*”

This comment elicited agreement among Latino/a participants, with many expressing how technology and social media messaging provide fewer intimidating opportunities to request information about PrEP use and available PrEP services.

### Normalizing PrEP messages outside the MSM community

Non-Latino/a participants emphasized how the increase of PrEP information in the whole community can help destigmatize negative attitudes towards PrEP use and the MSM community. Similarly in the LMSM group, one Latino/a participant shared a photo showing an informational poster about PrEP that appears to be on a wall in a bathroom, and stated:

“Simple, but effective just like PrEP. It might seem like everyone knows, but knowledge is power.”

This participant highlighted the effectiveness of disseminating simple PrEP information not targeted towards one specific audience or community. Another non-Latino/a participant shared a similar picture of a simple PrEP informational poster on a blank wall, and commented:

“PrEP access and materials should be available in all clinical/medical settings. The exposure would increase conversations and knowledge.”

This statement builds on the idea that basic PrEP information targeting people regardless of their race and sexual identity can help normalize HIV prevention. Both Latino/a and non-Latino/a participants identified public places (e.g., bus stops, public restrooms) and medical facilities as appropriate settings for distributing materials aimed at increasing PrEP knowledge and combating stigma. A non-Latino/a participant shared a picture of a large advertisement on an electronic public advertisement board and stated:

“Seeing information about PrEP in a very public area like the airport makes me feel like taking PrEP is part of a normal life. Presence in public spaces, not only where gay men live or hang out typically helps reduce the stigma of taking PrEP”.

**Fig 2:**
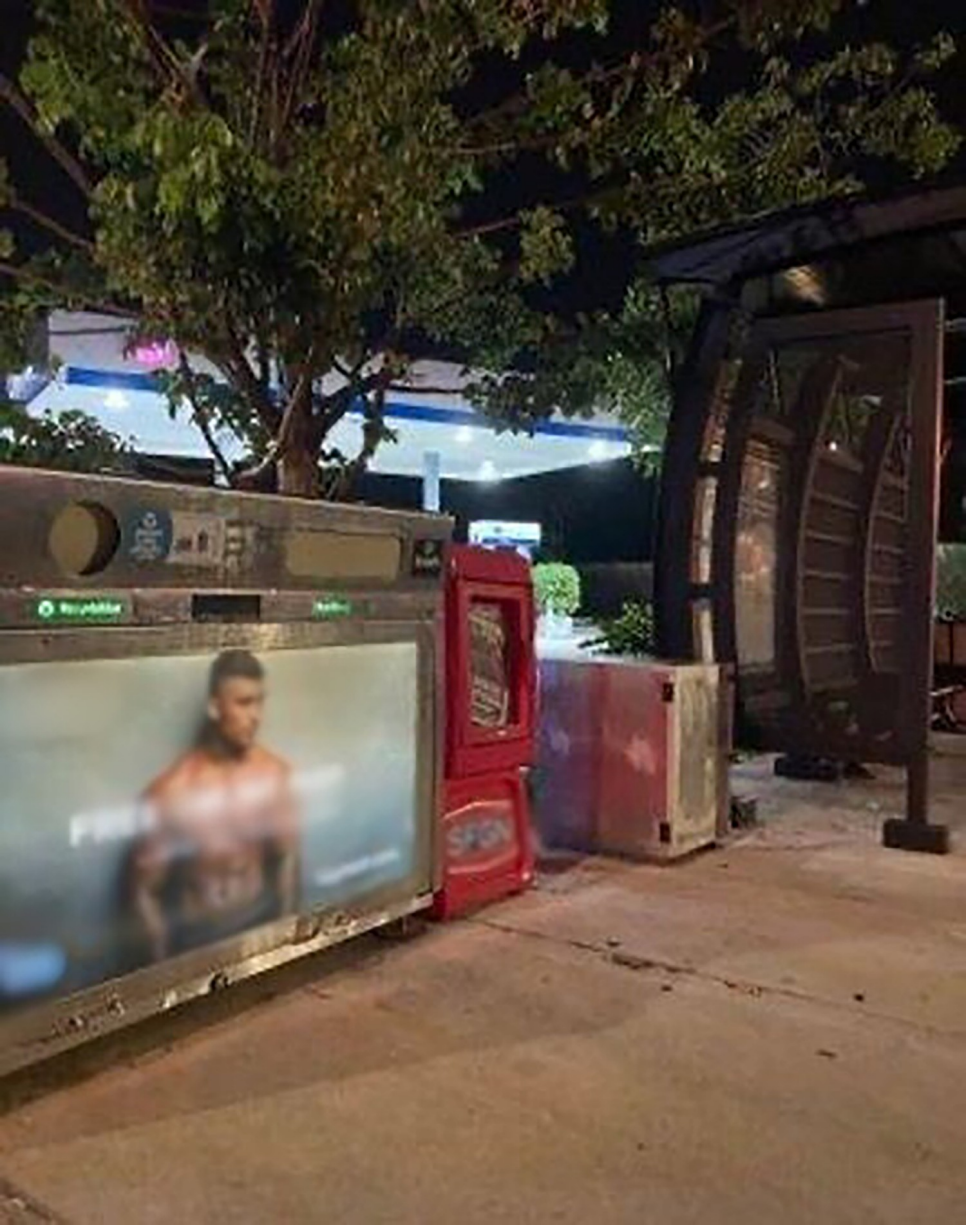
Photo shows PrEP advertisement next to a bus stop.

Another non-Latino/a participant shared a picture portraying a sign at a bus stop (Fig 2) and said:

“PrEP a double-edged sword, just like this image. On the one hand it attracts the attention of gay men by using a shirtless man in a gayborhood next to a bus stop, so it makes sense that it is targeted and that it is easily visible. On the other hand, it calls attention to sex and lust by only gay men, sending the message that this is a drug for gay men only because of our sexual activity compared to other demographics.”

This participant also mentioned that targeted PrEP promotion for the MSM community can reinforce negative stereotypes non-MSM communities have about MSM and recommended that PrEP campaigns should target communities at high risk for HIV using neutral and broader information to help normalize the use of PrEP in the general community. The need for normalizing PrEP messaging outside MSM audiences was mainly suggested by non-Latino/a participants.

### Access to knowledge and information

The theme of need for expanding PrEP knowledge–incorporating medical providers was identified as a barrier as part of the acess to knowledge snd information sub-construct.

Both Latino/a and non-Latino/a participants highlighted the need for PrEP information and access to PrEP services in the community. A LMSM participant stated:

“While there might be a lot of research done on the medicine, that isn’t knowledge that’s readily known by people who are trying to get on PrEP. We should expand this knowledge to the public”.

Latino/a participants expressed frustration regarding a disconnect between health care professionals and people who qualify for PrEP, which could lead to medical mistrust and misinformation. Relatedly, one non-Latino/a participant expressed:

“PrEP access and material should be available in all clinical/medical settings. This exposure would increase conversations and knowledge around its purpose.”

**Fig 3:**
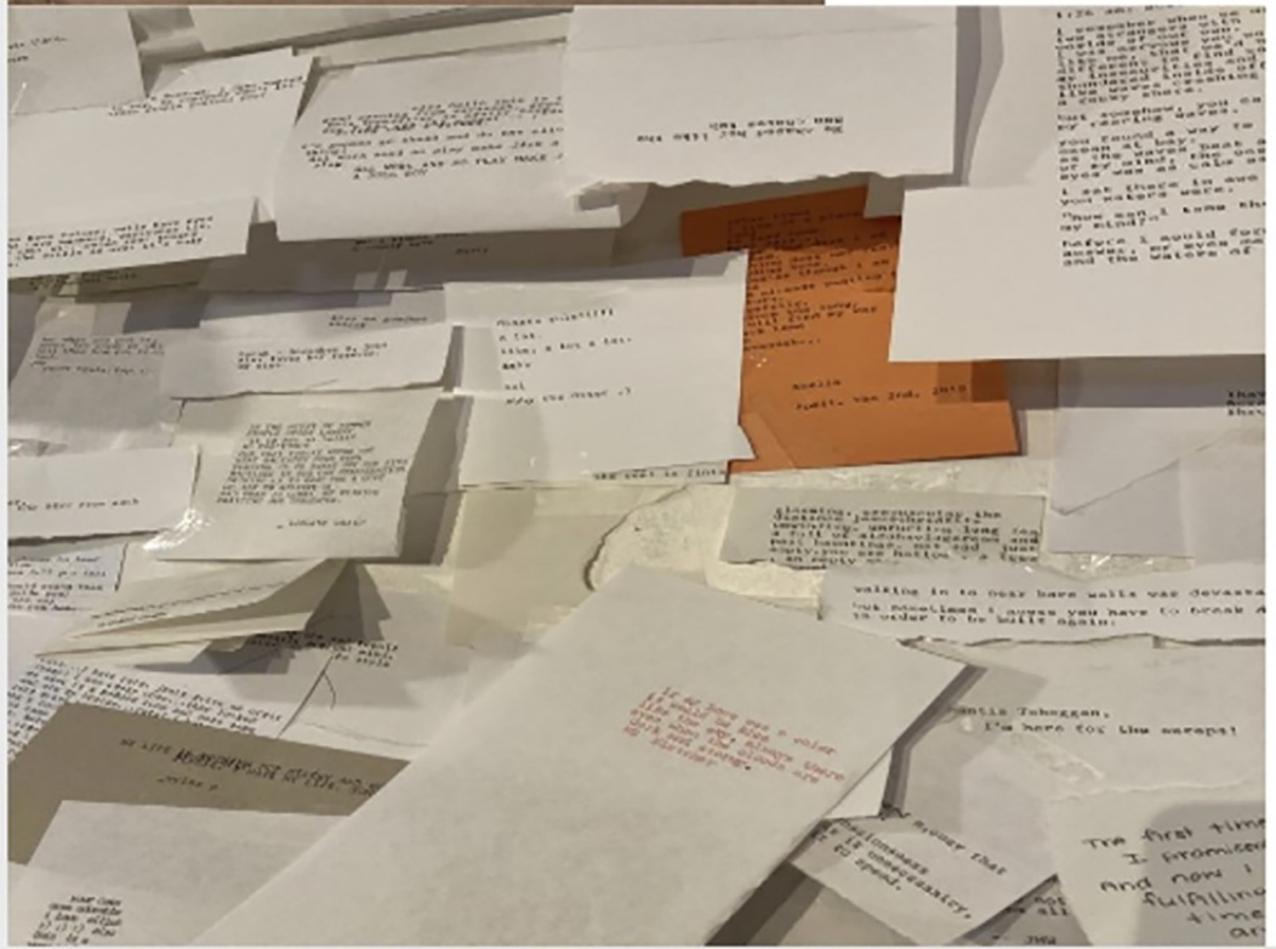
Photograph of scattered papers of different sizes.

Non-Latino/a participants highlighted the potential positive influence that physicians and medical providers could have in increasing PrEP initiation and uptake. Non-Latino/a participants expressed disappointment towards medical providers due to the lack of current training for initiating conversations about PrEP, for not facilitating access to PrEP, and for not being required to have knowledge and skills to prescribe PrEP. Latino/a and non-Latino/a participants echoed their desire for clear and simplified information regarding PrEP and PrEP initiation, and for trusted sources that provide this information. This sentiment was expressed by a non-Latino/a participant referencing Fig 3 below:

"Overwhelming—like the paperwork and bureaucracy to be able to get PrEP services. It also makes me think of information overload."

Participants indicated that PrEP education within patient-provider interactions will lead to expanded PrEP conversations within family and friends networks.

### Needs & resources of those served by the organization

#### Protection of self versus community

One of the central themes discussed highlighted the role that culture has on *why* and for *whom* PrEP was taken, which was found relevant to the CFIR domain of needs and resources of those served by the organization. Latino/a participants reported to be on PrEP not just to protect themselves, but to also protect others (i.e., for self and community). In contrast, non-Latino/a participants reported their motivation to be on PrEP as a personal choice to take care of themselves without reference to their community. Thus, for Latino/a participants, the use of PrEP was seen as part of their social responsibility—both protecting their community and protecting themselves were seen as being important. The importance of taking care of oneself, as well as taking care of one’s sexual partner(s), was echoed by several participants among Latino/a participants. A Latino/a participant expressed the following:

“Thinking of myself is also thinking of others. To what extent is the responsibility of not using condoms between partners if there is polygamous behavior?”

**Fig 4:**
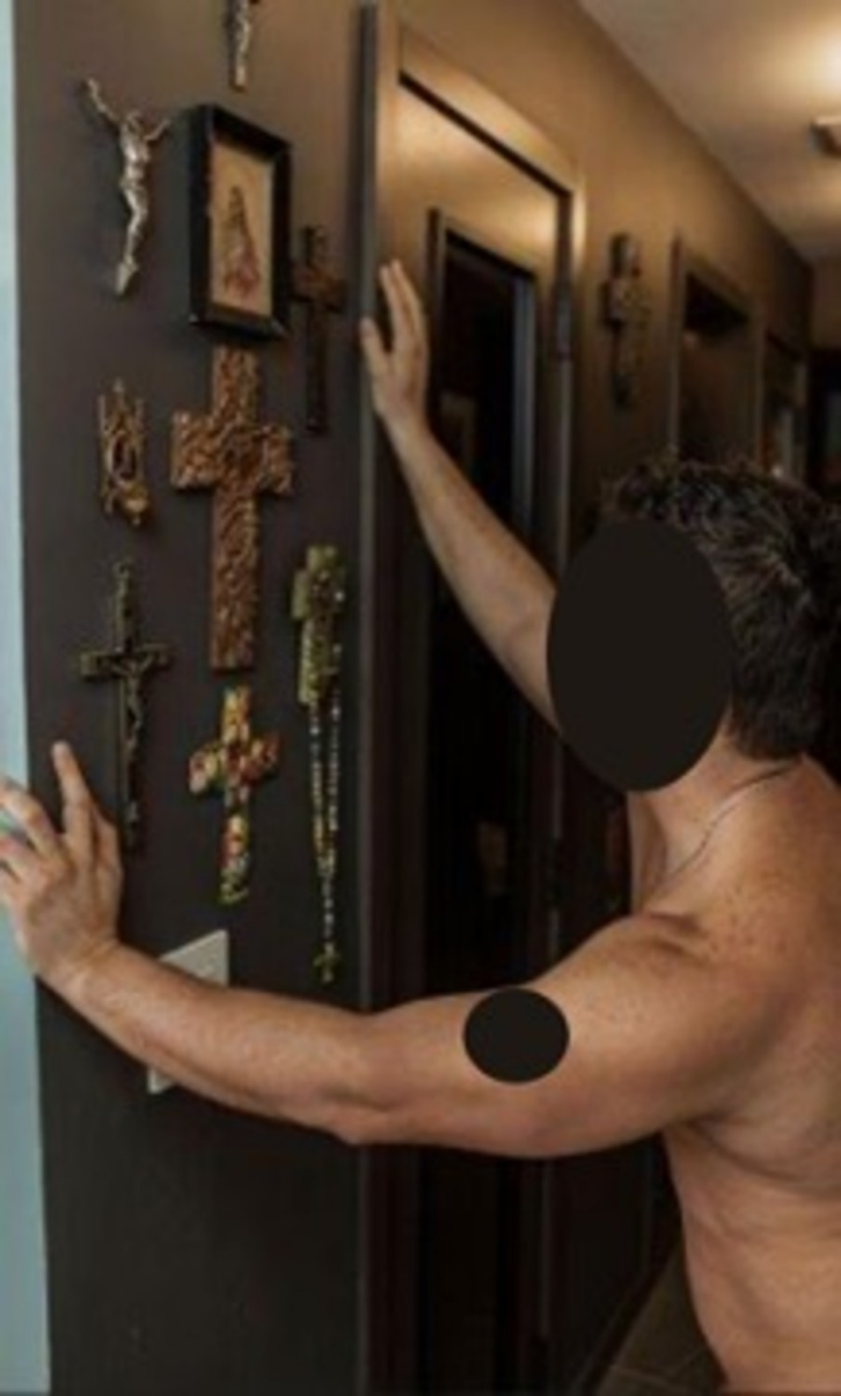
Photo of a man leaning with both hands on a black wall. The wall is decorated with several crucifixes and being looked at by the man.

As shown in Fig 4. religion was identified as important in Latino/a culture. Both religion and taking care of oneself and others were themes explicitly discussed by Latino/a participants.

Furthermore, as expressed in the following statement, in nuanced ways, religion can be linked with Latino/a MSM’s principle of social responsibility to protect others through the personal choice of taking PrEP:

“We still see how religion points at us and judges us but at the same time recognizes and includes us, this is something very positive in the community because it also invites us to take care of each other!”

Latino/a participants described religion as exclusionary yet embracing, judgmental yet unconditional. The above quote alludes to religion imposing judgement of non-normative sexuality while embracing individuals and encouraging them to care for themselves and others. Most importantly, this sentiment highlights the importance and centrality of religion for Latino/a participants.

In contrast, non-Latino/a participants mentioned that taking PrEP aligns with their personal priorities of protecting themselves, and as such it was important to match those priorities with actions—incorporating daily medication use into their routine. Additionally, non-Latino/a participants described their choice to be on PrEP as analogous to that of drinking responsibly; thus, once more emphasizing the power of personal choice and individual priorities.

Non-Latino/a participants expressed PrEP adherence as something they could embrace in their daily routines, such as during a coffee break. One of the photos taken by a non-Latino/a participant showed a coffee cup alongside a Descovy® bottle and a blue Descovy® pill in between. For some non-Latino/a participants, taking medication could be facilitated by coupling it with something that is already part of their daily routine to not forget to take their PrEP. This was expressed by the caption for that photo, which read:

“Coffee break can also be PrEP break. Just like many people make coffee a part of the daily routine, so can they incorporate PrEP into that routine.”

Some participants identified that their lack of setting priorities was a barrier for PrEP uptake because they did not have routines that could remind them to take PrEP. In addition, non-Latino/a participants emphasized the power of personal choice and aligning individual priorities with actions. One non-Latino/a member summarized the sentiments of his group by saying: “Just like a choice to drink responsibly. PrEP is a choice for one’s health.” This highlights the need for recognizing that PrEP is important for them, and why PrEP should be considered a health priority. Thus, non-Latino/a MSM depicted a pathway or explanation for why they take PrEP—concern for health and wanting to protect oneself (individual priorities) and choosing to take PrEP to protect one’s health (aligning action).

#### Available resource*s*

Within the inner setting domain, the sub-construct of available resources points to the important of structural influences including transportation, time, and money, which was identified as a key theme.

Having few PrEP providers and poor access to transportation were identified as factors limiting access to PrEP. One non-Latino/a participant stated:

"The location of PrEP clinics is far and few between. If the location of places that prescribe PrEP are more readily made available, more people would be getting on it. "

This comment generated an agreement among other participants who suggested that pop up parties PrEP can be used to distribute PrEP because MSM are unable to travel to one of the few locations where PrEP is provided. One participant stated:

“Transportation, some folks do not have a means to get to a clinic or testing center. Many folks ‘host’ but do not ‘travel.”‘

**Fig 5:**
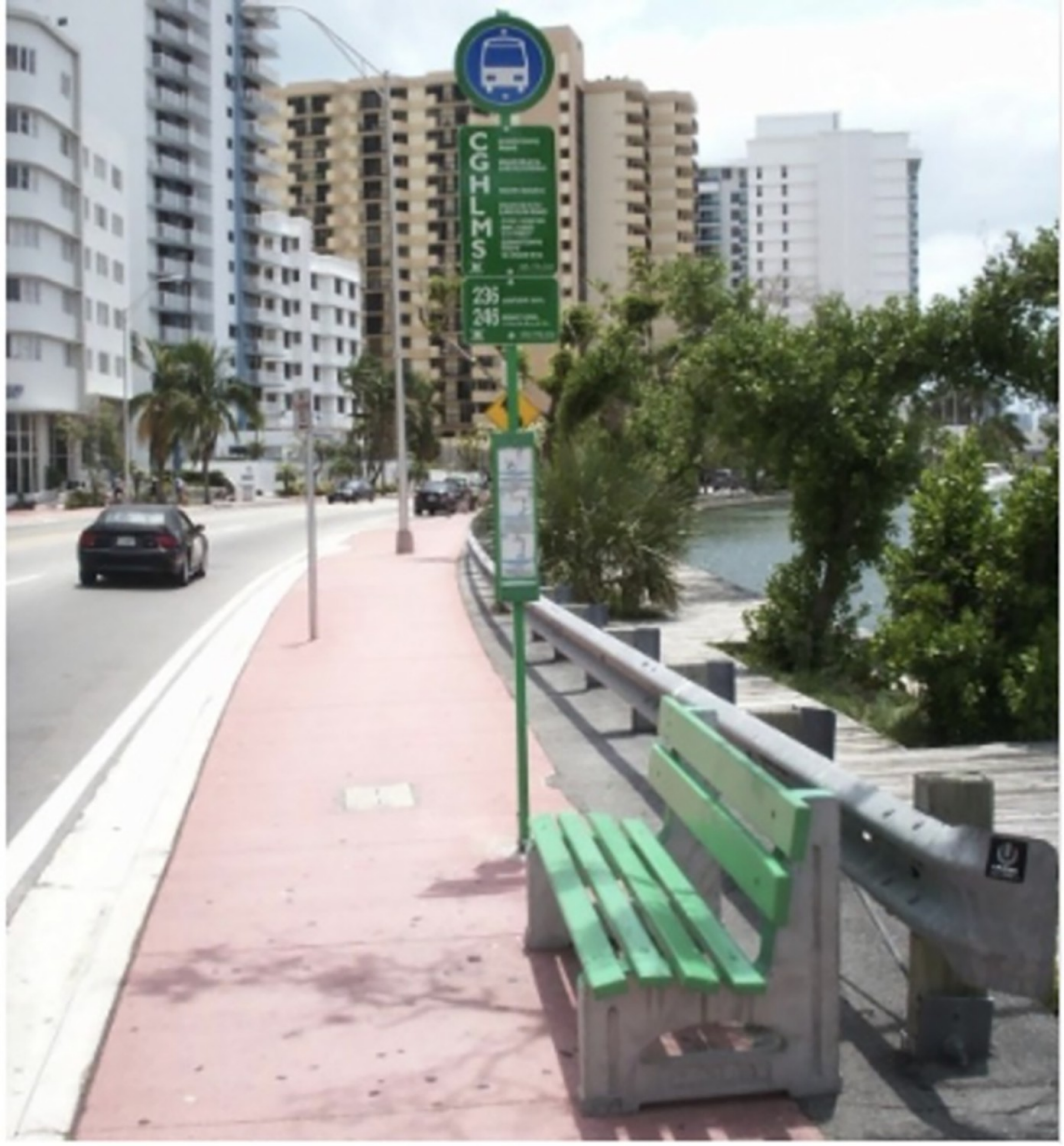
A photo of a bus stop presumably in the South Florida area.

For those without personal transportation (see Fig 5), inappropriate public transportation schedules and bad weather limited their access to PrEP services. A Latino/a participant explained:

“I believe transportation is a factor in how we can find a way to get help; unfortunately, it’s not easy especially knowing Miami weather it makes it super inconvenient to find public transportation; also, knowing that it’s not as convenient and may not take you to places near you at a short time period; places can be far away and may require more than one bus.”

For participants with personal transportation, high gasoline prices and heavy traffic were barriers for reaching PrEP services. A Latino/a participant mentioned:

“Time is money, and a lot of people don’t have time to set aside to pick up and organize how to receive their PrEP. I work a busy schedule and don’t always have the time to be able to go to a doctor’s appointment. We need more clinics that can meet after people’s work schedules let out”.

Having busy work schedules was also identified as a barrier for making appointments at a PrEP clinic. A participant advocated for more PrEP clinics offering after work services. A Latino/a participant mentioned that income and lack of health insurance were structural barriers to accessing PrEP. He expressed:

“Money is a barrier that keeps a lot of people from accessing PrEP. A lot of people don’t have health insurance and aren’t sure how to go about accessing a service like PrEP. We need to make sure PrEP is accessible to everyone, regardless of their income.”

## Discussion

The goal of this paper was to identify modifiable pre-implementation factors within CFIR domains, including the *outer setting*, *characteristics of the intervention*, and *characteristics of individuals*, for successful implementation of a PrEP program with participants of diverse racial/ethnic and gender identities. In regard to *outer setting*, we identified the need for normalizing PrEP messages within the MSM community and outside the MSM community to dissuade the negative effect of stigma on PrEP use. PrEP stigma has been associated with the belief that PrEP users are more promiscuous and more likely to engage in condomless sex than non-PrEP users [34].

Among groups that do use PrEP, stigma can also negatively influence continued uptake of PrEP. PrEP has been described as a “party drug” for people engaging in high-risk behaviors, condomless sex and promiscuity [34], which often leads to rejection from potential sex partners [35]. For this reason, PrEP stigma has also been considered as a key reason for PrEP discontinuation [36]. PrEP stigma can by extension influence perceptions of PrEP candidacy. Prior work found lower perceived PrEP candidacy to be related to overall PrEP naivete of those eligible for PrEP [37].

In our study, structural barriers under the theme of “built environment” included poor access to transportation, lack of geographic proximity to PrEP clinics, lack of access to PrEP health centers outside of work hours, and financial burden. The finding of transportation as a barrier is consistent with research that has previously identified limited transportation as a barrier for accessing HIV prevention services [33]. Additionally, this study found that when transportation barriers were not factors, other factors such as limited-service hours, lack of health insurance and financial uncertainty were identified as other structural barriers for PrEP initiation. Beyond the initial financial burden in accessing PrEP, participants discussed the time burden of PrEP, that is the inability to find time for arranging and continuing PrEP [38,39]. These results support implementation strategies that take advantage of telehealth services for visits that do not require blood or other lab work, including mailing patients their medication [40].

In regard to the *characteristics of the intervention*, participants discussed the need for trusted individuals who can answer questions and concerns regarding PrEP, including medical providers. Uptake of novel practices in HIV prevention generally relies on the transmission of information between people or organizations [41]. In our study, access to PrEP information by Latino/a participants was lacking in all social settings, while for non-Latino/a participants PrEP information was lacking in medical settings. Our findings highlight the lack of PrEP information provided by physicians or in healthcare settings and add to a growing body of literature that has identified inadequate PrEP or cultural knowledge among physicians and culturally non-responsive healthcare settings as barriers to PrEP initiation [42,43]. At the physician level, barriers to PrEP initiation included lack of PrEP knowledge, negative personal beliefs, lack of training, lack of clarity on who might be an appropriate candidate, and concerns about insurance coverage [44,45]. Prior work identified that healthcare system-level barriers include lack of communication about PrEP, funding for PrEP, and access to PrEP.

We also identified an association between perceived social responsibility and PrEP use; however, our findings indicated that ethnicity is a key determinant. Latino/a participants believed that PrEP use was their responsibility to protect the community and non-Latino/a participants spoke of the need to protect oneself. Prior research with Latino/a MSM found that the incorporation of cultural values can influence the success of health promotion programs [46]. These findings further the idea that utilizing the idea of community, religious or otherwise, could further the reach to Latino/a populations who could benefit from PrEP services [47]. For example, due to *religiosidad/espiritualidad* (a Latino/a construct that values religion), Latino/a undertake responsibility for their families and friends [46,48]. Previous research found that Latino/a who endorse these cultural values may be more likely to engage in PrEP conversations with members of their friendship networks [49,50]. In our study, non-Latino/a MSM associated PrEP use with a personal choice, prioritization, and action.

Strengths of this study include community engagement, the integration of the CFIR framework with the use of Photovoice and online focus groups to capture and prioritize individuals’ lived experiences. The CFIR was useful in developing the data collection measures (i.e., focus group guides) for the study as well as in developing the analytic framework for interpreting the qualitative data from the photographic data and focus group discussions.

Although the CFIR and implementation science methodology allowed us to identify implementation barriers and facilitators within specific domains, the findings also indicated that the CFIR may be limited in specifying the intersections among these domains that might reveal other culturally relevant factors influencing implementation. This study, for example, compared similarities and differences on determinants for PrEP use between Latino/a and non-Latino/a MSM. Similarities included the built environment (*outer setting)* as a barrier and the need for normalizing PrEP messaging to combat stigma. This comparison also allowed for a deeper understanding of personal drivers (*inner setting*) for initiating PrEP associated with how participants viewed their responsibilities to self or others. As noted earlier, non-Latino/a MSM were motivated to initiate PrEP as a benefit for themselves and Latino/a MSM considered it important to initiate PrEP to protect their community. This finding is important for understanding how *outer setting* and *inner setting* constructs might intersect to influence PrEP uptake.

Our work is not without limitations. We had a modest sample size of 24 and recruitment was aided by a community-based organization that offers PrEP, which may bias the responses and limit the generalizability to the broader South Florida community and beyond. Additionally, non-Latino/a participants consisted of a variety of racial, ethnic and cultural representation. Finally, social desirability could have also influenced our findings. Additionally, the use of CFIR may not adequately encompass cultural differences between researchers and participants. Despite these limitations, this study provided key findings upon which to formulate recommendations for potential implementation strategies and future research needs of Latino/a and non-Latino/a communities.

### Recommendations

A recommendation we propose regarding PrEP information at various levels developed from our consideration of the intersection between three of the CFIR domains that the research team identified—*individual characteristics* (e.g., perceptions of stigma, knowledge of and attitudes toward PrEP uptake) with *intervention characteristics* (e.g., simplicity in implementation) and *outer setting* factors (e.g., provider preparation and knowledge, aspects of the healthcare setting). Existing literature promotes PrEP research and education for providers with the goal of incorporating PrEP services in healthcare settings. PrEP information should be easy for people to understand from all education levels (providers as well as potential users) and come from a central source that is widely publicized. Such information should also be delivered using evidence-based practices such as the Office of Minority Health’s Culturally and Linguistically Appropriate Services (CLAS) Standards.

In selecting implementation strategies to move this work forward, implementers are encouraged to use the four themes we identified in this research to select strategies from evidence-based repositories as Expert Recommendations or Implementing Change (ERIC) strategies. Cultural adaptations should be developed in conjunction with implementation strategies to respect the cultural integrity of both the MSM community as well as the Latino/a community. For example, one of the identified themes was the need for normalizing PrEP messages within the MSM community and another was that there is an ethnic difference in how PrEP users might view their social responsibility (with non-Latino/a users’ needing to protect oneself versus Latino/a users’ needing to protect their community). Thus, PrEP messaging for implementation of a PrEP intervention may need to be tailored to align with cultural values of intended audience, whether that is for the MSM community, the LMSM community, or anyone at risk for HIV infection. Future research may be needed to better understand how to culturally adapt existing ERIC strategies that address the CFIR barriers identified in this research [51], while implementing PrEP best practices in culturally responsive and stigma-reducing ways.

This research also identified several factors in the *outer setting* domain, that independently of *individual* or *intervention characteristics*, might serve as structural barriers to implementation. Addressing these barriers may require policy-level changes within organizations or healthcare settings in order to improve the climate so that it adequately addresses patients’ needs. Additionally, organizations would need to decide that providing PrEP care is a priority and provide training or resources to increase provider knowledge and self-efficacy in delivery of PrEP and supportive services to potential PrEP users.

Future research is needed to identify constructs in other CFIR domains (e.g., *process*) to address other structural barriers and identify facilitators for successful implementation. Our findings suggested that partners other than the healthcare system may need to be engaged in implementation strategies as interventions are adapted for scale-up. These other partners might include public transportation systems and authorities such as gauging user need for medical transportation and then improving users’ access to travel to implementation sites or facilitating placement of ads and promotional messages at bus stops or on buses. Social services or other non-healthcare supportive services partners could also be engaged to disseminate trusted and reliable PrEP information and serve as welcoming implementation sites for PrEP interventions. Additional research could yield insights into how such factors as bolstering multi-sectoral partnership strategies, identifying champions from the MSM or LMSM communities, and focusing on other *process* domain facilitators identified in this research, could improve potential users’ access to and facilitate continuity of PrEP use.

## Conclusion

The integration of the CFIR framework with Photovoice and other qualitative methods provides an approach that can be applied successfully to yield key pre-implementation factors that inform cultural adaptations of existing evidence-based practices. Although we cannot expect to readily or easily change structural barriers to PrEP, we recommend that implementers consider systems-level components in their interventions as well as individual- or organizational-level components.

## Supporting information

S1 Table Mapped themes and CFIR constructs. (DOCX)
