# Supplementary material for: “PrEP a double-edged sword”: Integrating implementation science methodology with Photovoice to guide culturally-tailored pre-exposure prophylaxis (PrEP) programs for Latino/a and non-Latino/a men who have sex with men in South Florida
Source: PLoS One. 2024 Aug 9;19(8):e0305269. doi: 10.1371/journal.pone.0305269 (PMC11315311; doi:10.1371/journal.pone.0305269)
Supplement: S1 Table — (DOCX) [file pone.0305269.s001.docx]

| **CFIR Construct** | **Theme** | **Quotes** |
| --- | --- | --- |
| Knowledge & Beliefs about the Intervention | Normalizing PrEP messages within the MSM community | “Individuals in the community may not know their status because they do not ‘hook up’ or engage in sex with random people and because of this they do not get tested or take PrEP.” |
|  |  | “Access [to information] is everything. When opening your apps like Grindr, Scruff and others they advertise for different PrEP services in your community or online like Qcare+ or HeyMistr it makes the process seem more accessible and visible*” |
|  | Normalizing PrEP messages outside of the MSM community | “Simple, but effective just like PrEP. It might seem like everyone knows, but knowledge is power.” |
|  |  | “PrEP access and materials should be available in all clinical/medical settings. The exposure would increase conversations and knowledge.” |
|  |  | “Seeing information about PrEP in a very public area like the airport makes me feel like taking PrEP is part of a normal life. Presence in public spaces, not only where gay men live or hang out typically helps reduce the stigma of taking PrEP”. |
|  |  | “PrEP a double-edged sword, just like this image. On the one hand it attracts the attention of gay men by using a shirtless man in a gayborhood next to a bus stop, so it makes sense that it is targeted and that it is easily visible. On the other hand, it calls attention to sex and lust by only gay men, sending the message that this is a drug for gay men only because of our sexual activity compared to other demographics.” |
| Access to Knowledge and Information | Expanding PrEP knowledge – incorporating medical providers | “While there might be a lot of research done on the medicine, that isn’t knowledge that’s readily known by people who are trying to get on PrEP. We should expand this knowledge to the public”. |
|  |  | “PrEP access and material should be available in all clinical/medical settings. This exposure would increase conversations and knowledge around its purpose.” |
|  |  | "Overwhelming - like the paperwork and bureaucracy to be able to get PrEP services. It also makes me think of information overload." |
| Needs & Resources of Those Served by the Organization | Protection of self versus community | “Thinking of myself is also thinking of others. To what extent is the responsibility of not using condoms between partners if there is polygamous behavior?” |
|  |  | “We still see how religion points at us and judges us but at the same time recognizes and includes us, this is something very positive in the community because it also invites us to take care of each other!” |
|  |  | “Coffee break can also be PrEP break. Just like many people make coffee a part of the daily routine, so can they incorporate PrEP into that routine.” |
| Available Resources | Structural influences including transportation, time, and money | "The location of PrEP clinics is far and few between. If the location of places that prescribe PrEP are more readily made available, more people would be getting on it. " |
|  |  | “Transportation, some folks do not have a means to get to a clinic or testing center. Many folks ‘host’ but do not ‘travel.”’ |
|  |  | “I believe transportation is a factor in how we can find a way to get help; unfortunately, it's not easy especially knowing Miami weather it makes it super inconvenient to find public transportation; also, knowing that it's not as convenient and may not take you to places near you at a short time period; places can be far away and may require more than one bus.” |
|  |  | “Time is money, and a lot of people don’t have time to set aside to pick up and organize how to receive their PrEP. I work a busy schedule and don’t always have the time to be able to go to a doctor’s appointment. We need more clinics that can meet after people’s work schedules let out”. |
|  |  | “Money is a barrier that keeps a lot of people from accessing PrEP. A lot of people don’t have health insurance and aren’t sure how to go about accessing a service like PrEP. We need to make sure PrEP is accessible to everyone, regardless of their income.” |
